# On the Treatment of Strictures of the Urethra by Mechanical Dilatation, and Other Diseases Attendant on Them

**Published:** 1846-07

**Authors:** 


### On the Treatment of Strictures of the Urethra by Mechanical Dilatation, and other Diseases attendant on them. By *James Briggs,* Senior Surgeon of the Lock Hospital, &c. London, 8vo. pp. 62.

Having observed the advertisement of this little work on one or two occasions in the foul ward (*i. e.* column) of the newspapers, we were prejudiced against it as belonging to a class of publications which too frequently of late years have disgraced our profession; but an examination of its contents proves to us its localization there, was due to the mistake of the classifier of advertisements (for we suppose such a post must exist now these have so increased as almost to defeat each others object of attracting attention) and not to the wishes of the author. It is a purely professional treatise, and a very good one too.

*Dimensions of the Urethra.*—Mr. Briggs first alludes to the discrepant statements which have been made by different authorities as regards the length of the *urethra.* The mode in which he has endeavoured to determine this is by the passage of a catheter without a stilette, graduated in inches and fractions from a single aperture at its end. As soon as the urine commences flowing, we may observe the point of the scale which corresponds to the external meatus. In 60 persons the length was found to vary from $6\frac{3}{4}$ to $8\frac{1}{2}$ in. In three-fourths of the whole, the persons being of middle stature, it was between 7 and 8 in. The correspondence of the length of the urethra with the stature of the person is found to be sufficiently exact for all practical purposes; and the medium length of the passage may be stated at $7\frac{1}{2}$ or $7\frac{3}{4}$ in. Under all ordinary circumstances, the length of the canal does not undergo any material alteration in the distended or contracted state of the bladder. The length of its different portions will of course vary, as does that of the entire canal, in different individuals, but in the cast of an urethra $8\frac{1}{4}$ in. long, Mr. Briggs found the following to exist: from the orifice to the membranous part $6\frac{1}{2}$ in. and thence to the bladder $1\frac{3}{4}$. The casts which Mr. Briggs has made of the calibre of the urethra do not confirm the representations of this, contained in the plates of Home, there not being the sudden constriction at the junction of the membranous portion and bulb depicted in these.

"The portion of the urethra which extends from the apex of the prostate forward to a short distance beyond the arch of the symphysis of the ossa pubis, and in the natural state is the narrowest part of it, when distended, greatly exceeds the rest of the canal in its dimensions, and forms a large oblong sinus measuring from $1\frac{1}{4}$ to $1\frac{3}{4}$ in. in length, and in its transverse diameter at the broadest part from $\frac{10}{20}$ to $\frac{12}{20}$ in., the part of the urethra anterior to it not exceeding $\frac{7}{20}$ in. The broadest part of the sinus lies directly under the cartilaginous arch of the symphysis and above the bulb of the urethra, the latter of which with Cowper's glands lies over and conceals the membranous portion. The bulb is continuous or nearly so, with the prostate gland, and is covered by a dense fascia, which extends to the *erectores penis* muscles, there being little or no space between them, such as is usually artificially represented in dissections of these parts when the bulb is detached from the membranous portion and made to appear pendulous. The narrow part of the canal, as seen in these injections of the urethra, is at the point of union between the membranous and prostatic portions, *i. e.* immediately before its entrance into the prostate gland, where the two are united by a very small neck."

The *curve,* too, of the urethra comprises not only the portion of the canal usually assigned to it, viz. the bulb and membranous portion, but also the prostate and a portion of the canal anterior to the bulb "commencing, at about an inch anterior to the arch of the pubis, or the point where the corpus spongiosum becomes fixed, corresponding with the boundary marked by the anterior fibres of the *acceleratores urinæ,* the prostatic portion being rather more curved than the rest." The curvature is much greater prior to puberty ; but that of the adult urethra does not undergo much variation, and the degree of this which does exist seems to bear some relation to the stature of the individual ; for in persons of short stature, the sweep which an instrument makes in entering the bladder " is more sudden, and the handle carried lower than in those of large make, in whom it is oftener requisite to press the catheter back in a horizontal direction." Mr. Briggs denies the existence of *spasmodic stricture*, at least as dependent upon muscularity of the urethra. The *muscles* of the perineum encompassing the canal may at their points of insertion give rise to its compression, and impede the introduction of instruments. Then, again, the *lining membrane* of the urethra possesses remarkable *elasticity*, so that, on exmining the body of a child three years old, the author could scarcely obtain the admission of the point of a knitting-needle into this canal.

The directions given by the author for the *introduction of instruments into the bladder* are brief and judicious. He believes we best succeed with an instrument the curve of which is rather less than the natural curve of the urethra ; and much prefers placing the patient in the erect posture, as being that in which we can be most certain of the exact position of the point of the catheter. The weight of the instrument alone overcomes the resistance offered by the muscles surrounding the fixed portion of the urethra. It is more easily introduced by turning the concave portion downwards, care being taken that the point does not recede from the part of the canal it has reached, when the *tour de maitre* is made. All force is to be abjured, and if this is necessary to secure the onward progress of the catheter, we may be certain that its point is not properly directed. In the natural state of parts it is usually at about 5½ in. from the external orifice that resistance is met with, this being the point where the urethra becomes somewhat narrower, the direction of the canal changed, and the anterior edge of the levator ani inserted—circumstances also explaining the frequent occurrence of stricture here. If the point of the instrument on reaching the looser and larger portion of the urethra corresponding to the bulb is too soon elevated, or pushed forwards too hastily towards the narrower part, this looser portion of the urethra is carried forward on its point like a pouch, giving rise to the erroneous belief that the catheter has entered the bladder. Mr. Briggs does not believe this difficulty can generally be got over by drawing the penis forward on the catheter, introducing the finger into the rectum, &c., and that such manœuvres are unnecessary, as the point of the instrument may be adjusted to any angle we wish by due management of the handle ; the error indeed generally consisting in our raising this before it has entered the fixed or ascending portion of the urethra. Repeated attempts only irritate the parts, and, if the necessity of the case be not urgent, their repetition had better be deferred.

*Treatment of Stricture.*—Mr. Briggs disapproves of the treatment by caustic in any case, believing effective dilatation sufficient. He considers the common wax bougie and other flexible instrument as much less manageable and easily directed than an inflexible one, except indeed the stricture be situated at the anterior part of the canal. Moreover in its passage its point often becomes entangled and irritates the canal when the resistance of this is endeavoured to be overcome. Although not a proper means for treating stricture alone, its flexibility often renders it a useful auxiliary. Mr. B. has long been in the habit of using a succession of wedged-shaped sounds.

"The conical part of the instrument is about an inch in length, extending from the bend to a short distance from its point, the rest being of equal thickness. It has a definite curve, and is ground on each side, so as to leave an obtuse edge or projecting line running on the concave side, from within half an inch of the point to the part where the curve terminates. The extreme point to the extent of a quarter of an inch is uniform. The point of one instrument corresponds in size with the thick part or stem of the next, or is somewhat less. The difference between any two of the instruments in thickness, or between the point and stem of each, is about $\frac{1}{48}$ inc. or $\frac{1}{4}$ of the French line: the point of the smallest of these instruments measures in thickness $\frac{3}{48}$ or $\frac{1}{16}$ in., the stem $\frac{4}{48}$ or $\frac{1}{12}$ in.; that of the largest instrument $\frac{14}{48}$, the stem $\frac{16}{48}$, or $\frac{1}{3}$ inch. The series consists of 10 or 12 such instruments, but provided the principle of proportion in their construction be kept in view, the number or precise dimensions are, I think, immaterial.

"By giving an obtuse edge to a part of the curve of the instrument, which by the flattening of the two sides is nearly triangular, less resistance is made to its passage through the stricture, and a dilatation of the part is far more readily effected than by the mere conical sound; and more especially in the denser kinds of stricture, the removal of which cannot be effected without great perseverance in the ordinary and tedious process of treatment by the common bougie. * * * * * * * * * * By the introduction of such instruments in almost uninterrupted succession, the contracted part of the urethra may often be enlarged, almost at pleasure, to the extent of the rest of the canal, and very often at one time, especially where the stricture is confined to a very small portion of the canal, with as little pain as the patient would experience from a single introduction of a full-sized bougie."

These instruments, intended to produce the enlargement of the narrowing, cannot, however, be employed until the exact situation of the stricture is ascertained and a passage through it effected. To attain this, a very small bougie should be passed down to and pressed gently but steadily against the stricture, or if this is unsuccessful, a fine steel sound of uniform thickness ($\frac{3}{48}$ inch) is to be similarly used. In cases of difficulty, the attempt should be made immediately after the patient has urined. By these means, in a great majority of cases, the stricture can be penetrated, and even where this takes place only to a very slight extent the urine flows easier, and the instrument will pass on the next occasion. Where the stricture is so narrow as not even to admit the point of the finest wax bougie, Mr. Briggs has occasionally passed a whalebone bougie, with a point scarcely larger than a bristle. "In order to fit the whalebone for this purpose, it is necessary to soften it in warm water, and keep it for some time in a catheter, by which it will acquire a permanent curve." When once the stricture has been in this way passed, the author believes the contracted urethra may be enlarged by the conical instruments before-mentioned at option. Of course the number of times these may be required, as also their gradation, must depend upon the extent and closeness of the stricture; and one good piece of advice is given, viz.—that a smaller instrument should first be introduced at a sitting than the largest one which had been introduced on the previous occasion. When the instrument is firmly grasped and removed with difficulty, it is better to allow a few days to intervene before repeating its introduction; and it is not desirable to allow it to remain within the urethra more than a few minutes on each occasion.

Stricture is most commonly met with about $5\frac{1}{2}$ inches from the orifice, or at the point nearly corresponding with the arch of the symphisis. It occurs less frequently at about $3\frac{1}{2}$ inches near the root of the penis, being much more commonly cartilaginous in this situation. It is here, too, more tedious in cure, more liable to relapse, and oftener attended with incontinence of urine. Straight catgut bougies are often advantageously employed in its management here; but the cure is much expedited by the use of the wedge-shaped wire sound already described.

We may conclude our notice of this work by extracting a few observations upon the management of *retention of urine from stricture.* After observing that retention usually occurs from inflammatory or spasmodic action being superadded to the mechanical obstruction requiring general measures, as bleeding, warm-bath, opiates, and large doses of calomel for its relief, Mr. Briggs continues:—

"In old and confirmed strictures the part is generally so much contracted as to render the introduction of a catheter even of the smallest size rarely practicable. The only means which seem to promise success are that of passing a fine bougie down to the contracted part, and endeavouring to force the point of it within the aperture. If this can be effected, and the instrument suffered to remain for some time, on its removal the urine will sometimes be discharged in a very small stream, or by drops; but at each successive effort a small quantity will be voided sufficient to relieve the most distressing symptoms, and by a perseverance in the same measures, the stricture may be gradually enlarged, and the necessity of puncturing the bladder avoided. Where the opening of the stricture deviates, as is often the case, from the line of the urethra, a fine bougie possesses an advantage over every other instrument, in adapting itself to the irregularity: but in proportion to its fineness it becomes more liable to bend on the slightest resistance, and when the point has entered the contracted part, the waxy coating is ruffled up so as to prevent its advancing further. The instrument proposed (the fine steel sound) is not only free from the latter disadvantage, but, by uniting the two qualities of firmness and tenuity, gives the operator a command over it which he cannot have upon soft or flexible instruments. On this account the two kinds of instruments will be often found subsidiary to each other, and more useful than when employed separately. In cases where the stricture has been so narrow as not even to admit these instruments, I have more than once succeeded with a fine curved whalebone bougie, so tapered that the point has scarcely exceeded the size of a bristle.

"The notion of the absolute necessity of introducing a catheter into the bladder in these cases, leads often to ineffectual attempts to perform what is impracticable, and thereby to render the use of other means more difficult and uncertain. The simple introduction of an instrument, however small, through the obstructed part, when the retention arises from this cause, will, I believe, be found sufficient to procure a flow of urine, and to relieve the dilatation of the bladder, and thereby allow time for the dilatation of the stricture. And it is worthy of observation, that, however small the quantity may be which is voided, it will, *pro tanto,* take off the distension of the bladder, and consequently the straining and spasmodic pain, almost as effectually as if the bladder had been emptied by drawing off the whole of its contents by means of the catheter."

Mr. Briggs does not of course intend his little work for a complete treatise on stricture, and hence there are many points he entirely passes over which would have yet furnished interesting subjects for comment.

His object is merely to state the means of relieving stricture which he has found most successful, and his observations seem to us useful and judicious. We cannot however agree with him in the entire proscription of caustic, and believe that the rapid cure he describes following dilatation by metallic sounds will seldom be found permanent. Moreover, we regard these fine wire sounds as dangerous instruments in the hands of any one who is not constantly exercised in their employment.

